# Obituary

**Published:** 1853-04

**Authors:** 


### OBITUARY.

We are called upon to lament the death of Dr. William E. Horner, for a long time Professor of Anatomy in the University of Pennsylvania. He died on the 12th ult., of Disease of the Heart.

Died, on March 15th, at Brush Hill, Dupage Co., Ill., Dr. D. W. Boyd, of Consumption, after a protracted Illness. He was very much respected by the neighborhood of which he has been a practitioner for many years.

